# Developing allied health professional support policy in Queensland: a case study

**DOI:** 10.1186/1478-4491-12-57

**Published:** 2014-10-08

**Authors:** Karen E Bell, Fiona Hall, Sue Pager, Pim Kuipers, Hayley Farry

**Affiliations:** Darling Downs Hospital and Health Service, PO Box 358, Taroom, QLD 4420 Australia; Allied Health Professional Leader (Workforce) Allied Health Professions Office, Australian Service and Clinical Innovation Division, Queensland Health, Brisbane, QLD Australia; Metro South Hospital and Health Service, PO Box 4096, Loganholme DC, QLD 4129 Australia; CFAHR, Metro South H&HS and Population and Social Health Research Program, Griffith Health Institute, Griffith University, PO Box 6053, Buranda, QLD 4102 Australia; Darling Downs Hospital and Health Service, Cunningham Centre, Private Mail Bag No.2, Toowoomba, QLD 4350 Australia

**Keywords:** Professional Support, Policy, Evaluation, Retention, Allied Health

## Abstract

**Introduction:**

Evidence suggests that professional support for allied health professionals contributes to improved clinical practice, better client outcomes, enhanced workplace satisfaction, increased workplace morale and better clinical governance within organizations. Despite these benefits, the uptake of formal professional support is surprisingly low and implementation often *ad hoc*. Further, research investigating the development, evaluation and outcomes of implementing policy to establish such support is limited.

**Case description:**

Queensland Health has developed an organization-wide approach to supporting allied health professionals through a Professional Support Policy and guidelines. The processes of development, implementation and the evaluation framework of this State-wide Professional Support Policy are described. An evidence-based Professional Support Policy that is structured, collaborative and well evaluated will have benefits for allied health professions. However, policy introduction cannot occur in isolation. Current practice does not follow current evidence in the area of professional support implementation. This study describes a current practice baseline for participation prior to the mandating of such a policy. There is a need for improvements in participation rates, documentation and capacity building.

**Conclusions:**

A workforce policy with broad scope should increase the access to, and consistency of, professional support to allied health practitioners. Such policy should facilitate a higher quality clinical practice, better client outcomes, enhanced workplace satisfaction and morale. It may also maximize the recruitment and retention of allied health professionals. Mandating policy should see participation commensurate with that policy. A future step will be a Post Policy Implementation Review to determine the success and effectiveness of the Professional Support Framework within Queensland Health.

**Electronic supplementary material:**

The online version of this article (doi:10.1186/1478-4491-12-57) contains supplementary material, which is available to authorized users.

## Background

Professional support refers to activities that create an environment where personal and professional growth may occur [1] and includes professional supervision. It is described in the literature as contributing to the clinical governance of organizations and enhancing the professional development of individual clinicians [2, 3]. Within Queensland, two reviews of a high profile incident resulting from a breakdown of standards and safety of hospital care were highly critical of clinical governance and culture within the organization. This resulted in organizational restructure and the establishment of a new multifaceted clinical governance framework described as a ‘web of policies, processes and accountabilities’ [4]. One aspect of the resulting changes was a substantial improvement to the provision of professional support for the allied health (AH) workforce within Queensland Health.

The literature contributing to our understanding of professional support within the workplace predominantly focuses on one aspect - supervision. In their literature review of AH clinical supervision policy in Australia, Fitzpatrick *et al*. argued that clarity of definition is an essential first step in policy development [5]. Therefore, gaining an understanding of what high quality clinical supervision is, and how it is best put into practice, is the first step in developing supervision policy [5]. As such, the need was identified for policy characterized by a collaborative, supportive approach, with well-defined criteria and a ‘whole of health’ model. Such policy reflects an ‘agreed aim and definition of supervision; a consensual understanding of what constitutes effective clinical supervision; high-quality training for clinical supervisors; and, clear implementation mechanisms and a united model of clinical supervision’ ([5] p 464).

The ideal dimensions of policy implementation in the area of professional support (including supervision) nationally and internationally are not clear. In the mental health nursing context, Rice *et al*. [6] recommended a focus on policy that includes the processes of contracting, provision of time within the working day, clear managerial commitment to the process, completion of appropriate training as well as a process for monitoring and evaluating the impact. In a recent systematic literature review of strategies to boost recruitment and retention of nurses in aged care, the provision of ongoing supervision and education were identified as essential [7]. The review included several studies which detailed the importance of providing professional support, and, more specifically, mentoring, peer support programmes, and clinical supervision.

To date much of the literature in this area relates to the very large nursing workforce, to single discipline experiences, for example, within social work or psychology [8], or to smaller multidisciplinary groups such as occupational therapy, physiotherapy, podiatry, psychology, social work and speech pathology as described by Dawson *et al*. [9]. It is generally also confined to supervision as the means of professional support.

Government policy may be seen as an endorsed position evidenced by legislation, formal government documents/strategies, or industrial agreements [10]. As such ‘policy’ has facilitated effective development and utilization of the health workforce in areas including health promotion and prevention, for example, drug and alcohol use [11] and responsible driving [12]. However, the impact of policy development in the area of professional support (including supervision) nationally and internationally is not so clear. The current case study seeks to contribute to this literature with a broader focus on the large multidisciplinary AH workforce, and by reporting on the development, implementation and evaluation of professional support policy using several types of support.

### Case description

This case study describes the development, implementation and evaluation framework of the Queensland Health State-wide AH Professional Support Policy. Queensland Health employs close to 5,000 professionals from 16 discipline groups including audiology, exercise physiology, clinical measurement sciences, medical radiation professions, music therapy, dietetics, nutrition, occupational therapy, orthoptics, pharmacy, physiotherapy, podiatry, prosthetics and orthotics, psychology, rehabilitation engineering, social work and speech pathology. It has a geographically dispersed network of more than 178 hospitals and health care facilities, with 15 health service districts covering a range of service models across the continuum of care.

The Professional Support Framework includes professional supervision, peer group supervision, mentoring, in-service training, journal clubs, peer review and work shadowing as potential support activities (definitions contained at the end of Additional file 1). It includes policy, supporting guidelines, training materials and resources for implementation. The Professional Support Policy and guidelines recognize that professional support is central to promoting the personal and professional development of AH professionals, and that it has a consequential impact on consumer safety and quality, effectiveness and dependability of services, and retention of staff.

**Figure 1:**
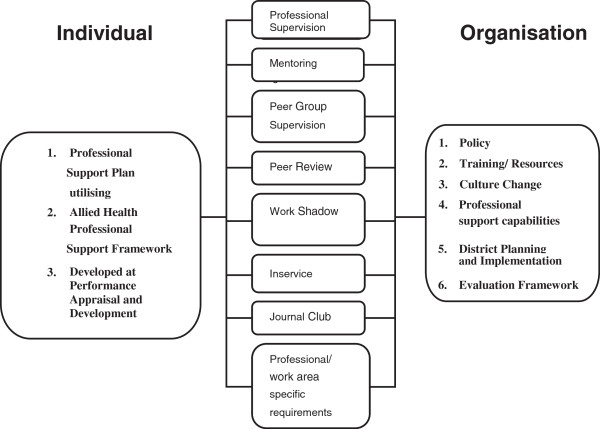
**Professional support framework.**

This Professional Support Policy document guides AH professionals and their managers regarding the minimum standard of professional support activities in which Queensland Health would expect staff to be engaged. The Professional Support Policy is supported by the Professional Support Framework to ensure well-developed criteria, an evaluation framework and resources (including guides and training packages) to support the sustainable implementation of the Professional Support Policy. A detailed description of the framework is documented elsewhere [13]. In the current case study, the methodology used in development of the Professional Support Policy and guidelines is described. Figure 1 demonstrates the components of the framework.

### Developing the policy

#### Organizational drivers

The opportunity for policy development in this area resulted from a number of factors including organizational restructuring, the expansion of the corporate arm of the Allied Health Unit, some centralization of responsibilities, the push for clinical governance reform, and the availability of human and financial resources required for developing and driving a supportive and collaborative approach to a workforce professional support policy. A centralized workforce unit was responsible for overarching policy with capacity to govern and fund workforce initiatives. This was supported by large groups of representative AH professionals and resources to undertake a collaborative approach. As a part of the approach a project team was funded and recruited to develop policy, provide implementation support, develop and deliver training and to evaluate the outcomes of the project with a comprehensive evaluation framework.

#### Literature review

Historically, the workforce had been supported by a variety of district or local approaches to professional support. The first step in developing a state-wide coordinated approach to policy development was a review of the evidence and current practice within the organization. The literature review conducted prior to the development of the policy noted that professional support contributes to high quality clinical practice among AH professionals, better client outcomes, enhanced workplace satisfaction, increased workplace morale for individuals [14] and better clinical governance within organizations [4]. It found that despite the well-known benefits, the uptake of professional support may be low [15, 16] and implemented in an *ad hoc* fashion [14], with many supervisees identifying that they are not given the opportunity to undertake supervision [17]. The review noted that the implementation of supervision requires organizational support to help market the concepts and benefits of supervision and increase participation [18]. Indeed, successful implementation of professional supervision relies on organization time and resources [19], since organizational issues, such as lack of choice around a supervisor, have been found to be key barriers to effective supervision [20]. The literature relating specifically to policy has highlighted the importance of agreed definitions, consensus on elements that contribute to effectiveness, including training and clear implementation mechanisms [5] as well as policy that includes the processes of contracting, organizational commitment to the time required, and a process for monitoring and evaluating the impact [6]. The literature also highlighted the lack of policy outside those developed by discipline-specific professional bodies, including a lack of policy that discussed means of implementation [5].

#### Consultation and review

On the basis of the literature review and other organizational policy (including practice supervision in mental health) a draft Professional Support Policy incorporating the elements identified in the literature was developed. Consultation utilizing teleconferenced open-ended feedback on the policy occurred through an internal steering group. This group was able to widely consult throughout the workforce utilizing extensive state-wide consultation with AH networks, directors of AH in all of the districts, with discipline representatives, unions, workforce development officers and training and education staff. This consultation included specific forums and workshops with stakeholders using prepared presentations and gathering of group feedback. Extensive consultation was conducted across more than 12 District Health services, drawing from more than 16 AH professional groups. The diversity of professions, service models and locations necessitated a broad, but flexible policy. The draft policy and implementation guidelines that supported the policy were then published internally on the Intranet for written feedback from the entire AH workforce. A total of 44 individual and group submissions were received providing feedback, which was then integrated into the final policy presented for endorsement. This version of the policy is available in Additional file 1.

Early challenges in the policy development process included the difficulty of developing consensus around the definition of professional support options that would be included, the selection of activities for inclusion, and the establishment of minimum standards across a diverse workforce. The scope of the endorsed policy included 16 AH professional groups participating in at least some professional supervision, peer group supervision and/or mentoring, with other activities encouraged.

#### Policy essentials

The minimum requirements for participation in professional supervision or peer group supervision or mentoring are as follows:

-  Newly graduated’ AH professionals (less than two years full time practice since obtaining qualification) shall participate in a minimum of one hour of per week or equivalent
-  AH professionals who have practiced for the equivalent of between ‘two and five years’ in a full time capacity shall participate in a minimum of one hour per fortnight or equivalent
-  AH professionals who have practiced for the ‘equivalent of five years’ or more in a full time capacity shall participate in a minimum of one hour per month or equivalent

AH professionals and their managers are required to jointly determine where additional professional support is required to meet legislative, professional or individual requirements. Furthermore, the policy requires that at least 50% of professional support is to be obtained from an appropriate supervisor or mentor within the same profession. To accommodate the diversity of professional groups, the policy and set of supporting guidelines are written in a way that provides flexible options for professional support. However, they are implemented in a way which was structured and evaluated through the existing mandatory Performance Appraisal and Development Plan or a formalized professional support tool.

The policy outlines responsibilities for providing professional support. That is, where appropriate, all AH staff with greater than two years’ experience are expected to provide professional support. A determination of appropriate amount is made between the AH staff member and their manager. In addition, profession-specific managers were consulted in determining the appropriateness of staff to undertake professional support provision. All supervisors and mentors have access to support in their provision of supervision/mentoring and professional support activities. AH professionals and their managers are required to consult with profession specific managers in determining the requirement for, and appropriateness of, staff to undertake professional support provision.

The professional support guidelines operationalize the policy and support implementation. The guidelines include definitions of professional support, principles of the Professional Support Framework, how to set up a Professional Support Plan and an overview of the components of professional support. The guidelines elaborate on the minimum requirements for professional support, fully describe the professional support resources available and provide suggestions for undertaking professional support documentation including provision of templates such as supervision, mentoring and peer group supervision agendas, agreements, records and evaluations. The guidelines also define the role of supervisor/mentors, describe difficulties that may arise and how to deal with them. The guidelines are available from the corresponding author on request.

#### Baseline survey

A Professional Support Evaluation Framework was developed and used to obtain base-line data to evaluate the uptake and implementation of the State-wide Professional Support Policy. Requests to participate in a pre-policy survey were sent to all directors of AH, professional support ‘champions’, workforce development officers, clinical educator managers and discipline chairs to distribute to their networks. Approximately 5,000 AH Professionals were asked to complete the online questionnaire. The 28-item survey took approximately 5 minutes to complete and responses were anonymous.

Beyond demographic information, the survey assessed current levels of participation in forms of professional support and in the Performance Appraisal and Development processes. It also looked at documentation use as well as proportions of discipline-specific support. There were seven questions relating to actual participation in professional support in the preceding three months. The survey ended with questions regarding the amount of supervision of professional support functions and training undertaken. The survey is available from the corresponding author.

**Figure 2:**
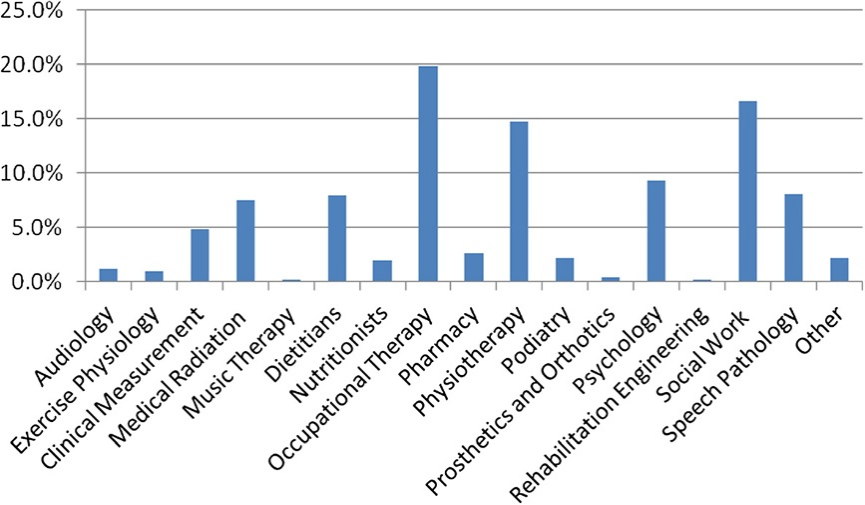
**Survey participants according to profession.**

**Figure 3:**
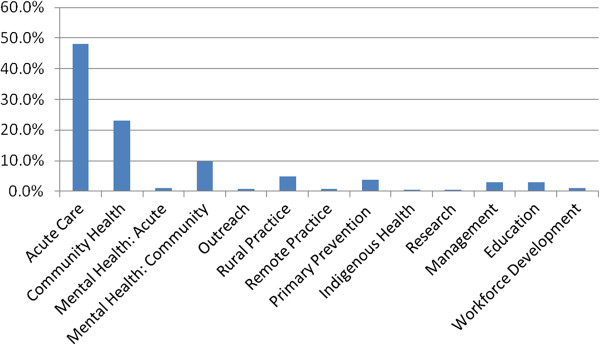
**Participation according to service area.**

One thousand five hundred and thirteen AH professionals participated, representing approximately 30% of the workforce from the 16 professional groups eligible to participate. Figures 2 and 3 provide an illustration of the diversity of participants across professional group and service type. The majority of participants were from an acute care or community health setting; other service areas included rehabilitation, community mental health, rural practice, primary prevention, health promotion, public health, education and management. Thirty-four percent of respondents were from metropolitan facilities and 5% from remote districts, the balance were from rural and regional areas.

The policy prescribes participation commensurate with level of experience from professional supervision, peer group supervision and mentoring. At the time of the survey, participation in a minimum of 1 hour per week for those with less than 2 years’ experience (14% of the respondents) was 11%, a minimum of 1 hour of formal supervision/mentoring per fortnight for those with 2 to 5 years’ experience (17% of respondents) 14%, and a minimum of 1 hour per month or equivalent for those who have practiced for the equivalent of at least 5 years (69% of respondents) 48%. Despite the expectation that all staff would participate, 13% of AH staff surveyed did not participate in any professional support activities in the preceding 3 months.

**Figure 4:**
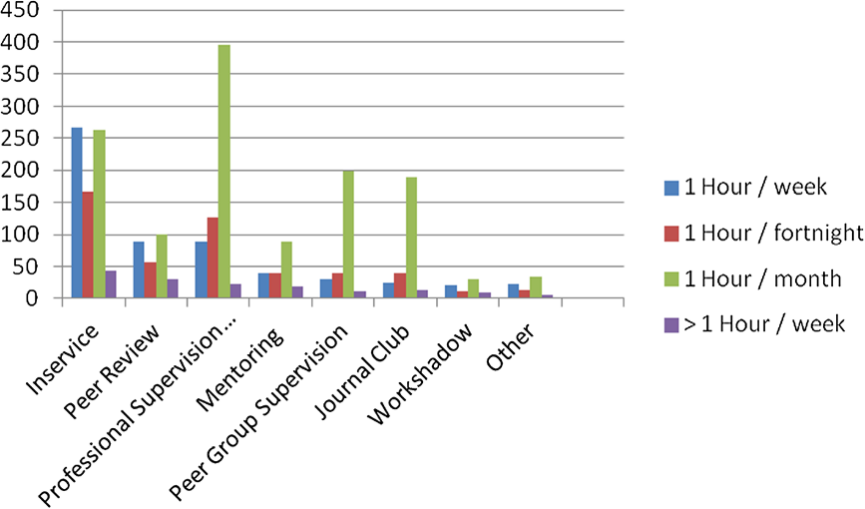
**Current participation in professional support.**

Acknowledging that staff could participate in more than one form of support: in descending order the attended professional support activities were in-service, professional supervision, peer group supervision, journal clubs, peer review, mentoring and work shadowing (Figure 4). Most of these activities were discipline-specific and intermittent.

**Figure 5:**
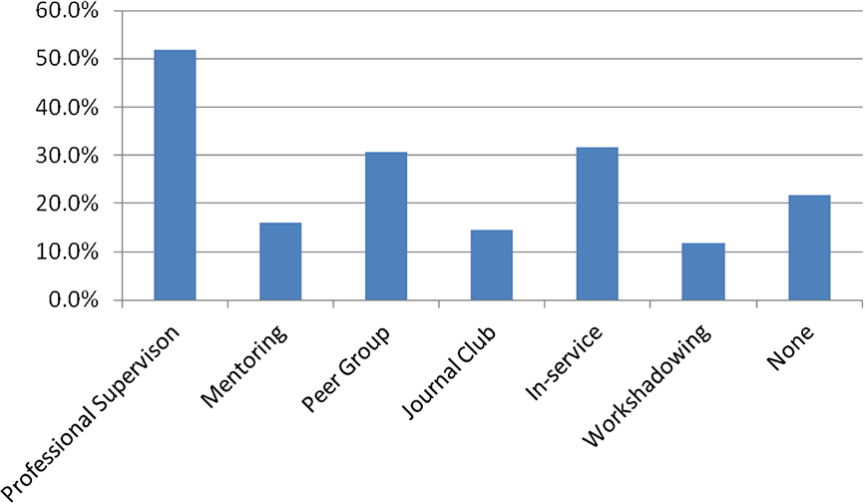
**Training in professional support.**

The pre-policy data collection also assessed current levels of participation in Performance Appraisal and Development (PAD) activities. Twenty-eight percent of staff did not have these mandatory plans in place. Further, 57% of staff did not have any formal agreement documentation for any kind of supervision, and 52% did not document their sessions. Twenty-one percent of staff had no training in any of the professional support options. Figure 5 shows that 81% had training in professional supervision and peer group supervision when combined. These were the major foci of the training offered.

### Implementing the policy

Based on the survey, the project team made recommendations to the Allied Health Workforce and Coordination Unit. An Implementation and Communication Plan was developed outlining a clear strategy to implement the Professional Support Policy. This plan provided strategies to engage AH managers, leaders and professionals and included memos, information sheets, ‘frequently asked questions’ sheets, state-wide videoconferencing, project staff engagement processes that facilitated enablers and address barriers to the local implementation. These strategies targeted those AH professionals and managers that were not aware of the policy requirements. Resources were developed by the training arm of Queensland Health and included training programmes, professional support policy guidelines, implementation tools and professional support guides. It was recommended that the resources should be actively marketed to support the implementation of the policy and that a 12-month post implementation review should focus on evaluating the success and effectiveness of the Professional Support Policy.

To facilitate workplace readiness, a state-wide multistrategy approach was taken to implement the Professional Support Policy. To reinforce accountability and responsibility for governance, strategic planning, and coordination of resources, the assistance of the policy and planning corporate arm of Queensland Health was enlisted. Mandating the policy demonstrates organizational commitment, supports the provision of ‘within work’ time allocation evidenced by the literature and integrates professional support into workplace culture.

Awareness of the policy and information about professional support and the Professional Support Framework were disseminated through flexible means such as teleconferencing, videoconferencing and face-to-face consultations. Leadership was delegated from the organizational level to local team initiatives to build workforce readiness, acceptance and enthusiasm. This facilitated the setting up of professional support plans, recognition of the minimum requirements for professional support, the use of professional support documents, the selecting and supporting of supervisors and mentors, and the capacity to deal with difficulties that arose.

The operational implementation of the policy was overseen by the State-wide Professional Support Reference Group. This group represented District Health AH directors, discipline directors, AH networks, AH managers, the AH Clinical Education and Training Unit, discipline-specific project officers, district professional support representatives and champions, workforce development officers and non-AH managers. This group facilitated engagement and monitored difficulties across the workforce, fostering local ownership and addressing emerging problems.

Professional support project staff supported the implementation of the policy in districts by coordinating training in professional supervision and peer group supervision, supporting district trainers and providing resources. Other learning and development programmes (such as training in the Performance Appraisal and Development planning process) facilitated implementation of the policy for AH staff across Queensland Health.

To support training in these areas, collaboration with State-wide Clinical Education and Training Units and workforce development officers in each district was established. All training, whether externally sourced or developed internally, provided online guides with associated training packages including suggested documentation for supervision, peer group supervision, mentoring, journal club, peer review in-service and work shadowing. Training was offered face-to-face and via videoconferencing. The training providing a consistent product throughout the workforce that facilitated ease of movement through the organization for staff was delivered through an internal registered training organization that ensured quality, consistency and evaluation.

The Post Implementation Review will include another survey of the workforce as well as interviews with AH professionals, line managers and clinical educators. Our expectation, following mandating of the policy, is that follow-up surveys will see an increase in total participation rates, with a frequency commensurate with degree of experience, and much improved documentation of professional support activities and participation in PAD. We will expect to see a move away from in-service as our most frequently utilized form of professional support and a move towards the more reflective professional supervision, peer group supervision and mentoring. Building capacity by increasing numbers of trained staff will also be expected. The introduction of this policy is hoped to result in more staff identifying Queensland Health as an employer of choice and to influence both recruitment to and retention within the organization.

## Conclusion

Implementation of policy requires significant cultural change, particularly in areas or disciplines where professional support has not previously been consistent. Culture change and mandatory policy in the organization should promote the use of professional support activities as a valued (and non-negotiable) part of work roles. In light of the low response rate, a key part of the policy implementation is about raising awareness of the policy itself. Establishment of the policy required strong organizational leadership to promote understanding of the importance and value of professional support and ensure its integration into the regular workload. It required support from directors of AH, AH team leaders and line managers, workforce development officers, clinical educators and professional support ‘champions’. In addition to management support, recognition of the extent of culture change is required, as is capacity building through training, the provision of resources, and dedicated staff to drive change, consult, collaborate and evaluate.

There are indications that working in a learning culture improves clinical services [21, 22] and that supporting clinicians maximizes morale and leads to greater retention [8]. However, to date there are no direct measures of the effect of policy implementation on retention rates or clinical outcomes. Our aim in this initiative is to increase the numbers of AH staff who are trained in, participating in, and effectively documenting their professional support activity. Further research with a Post Implementation Review of the framework, guides, training and policy will be undertaken within Queensland Health. The impact of the Professional Support Framework on morale (work climate), recruitment, retention and better clinical outcomes will also be explored and will be the subject of future publications.

The case study provides a description of our experience within QH of implementing evidence-based professional support policy. It provides details of rare multidisciplinary AH policy development including details of an implementation strategy that provided benchmark data for the provision of professional support prior to the policy implementation and an evaluation framework to assess the impact of the policy through the change management process.

## Electronic supplementary material

Additional file 1: **Professional support policy.** (DOC 103 KB)

## Abbreviations

AH: allied health
PAD: Performance Appraisal and Development.
