# Supplementary material for: Developing allied health professional support policy in Queensland: a case study
Source: Hum Resour Health. 2014 Oct 8;12:57. doi: 10.1186/1478-4491-12-57 (PMC4197267; doi:10.1186/1478-4491-12-57)
Supplement: Supplementary file 1 — Additional file 1: Professional support policy. (DOC 103 KB) [file 12960_2013_451_MOESM1_ESM.doc]

**Additional file 1: Professional Support Policy**

Policy #<insert number here>

**Allied Health Professional Support Policy - (BR 5.4)**

## Policy Statement

The policy provides the minimum requirements for participation in formalised professional support for allied health professionals.

It recognises that participation in professional support is central to the safety of patients/consumers and the quality, effectiveness and dependability of Queensland Health services and is mandatory regardless of clinical area, career stage, location or profession.

The Allied Health Professional Support Policy is one component of the Allied Health Clinical Governance Framework.

Professional support is a component of the Performance Appraisal and Development **(PAD) process.**

## Intent of this policy

- Safeguard and improve the safety and quality of patient care
- Maintain the highest possible standards of allied health practice, service and consumer outcomes through a formal process of professional support
- Maximise employee morale and retention

## Scope

The Policy applies to **Health Practitioners within the following allied health professions:**

- Audiology
- Exercise Physiology
- Clinical Measurement
- Medical Radiation Professionals (Medical Imaging Technology, Radiation Therapy, Nuclear Medicine Technology, Breast Imaging Radiography)
- Music Therapy
- Dietetics
- Nutrition
- Occupational Therapy
- Pharmacy
- Physiotherapy
- Podiatry
- Prosthetics and Orthotics
- Psychology
- Rehabilitation Engineering
- Social Work
- Speech Pathology

## Principles

**Professional Support Components**

All allied health professionals should participate in a minimum requirement of at least:

- Professional supervision or
- Peer group supervision or
- Mentoring

The option/options chosen must be implemented in a way which is structured and evaluated.

In addition, allied health professionals are encouraged to participate in other components of professional support, including peer review, journal clubs, in-services and work shadowing.

The policy recognises that the identification of and linkages with a profession specific manager for each Health Practitioner is central to the development of the safety, quality, effectiveness and dependability of Queensland Health services.

At least 50% of professional support is to consist of profession-specific support and is obtained from an appropriate supervisor or mentor within the same profession; the remaining 50% could be obtained from an Allied Health or non-Allied Health profession depending on work requirements.

Allied health professionals employed in Queensland Health’s Mental Health Services have a specific policy for Practice Supervision for Allied Mental Health and Clinical Supervision Guidelines for Mental Health Services that outline the minimum professional support requirements for mental health practice supervision. The allied health Professional Support Policy and Guidelines are intended to complement and provide additional options or supports for mental health allied health staff and do not replace the principles, definitions, or requirements as outlined in the mental health policy or guidelines for the mental health workforce.

**Minimum time requirements for Professional Support activities**

Application of this policy must be consistent with the following principles. The *minimum requirements* for participation in professional supervision or, peer group supervision or mentoring are as follows:

- Newly graduated allied health professionals (under two years full time practice since obtaining qualification) will have a minimum of one hour of formal supervision/mentoring per week or equivalent
- Allied health professionals who have practised for the equivalent of between two and five years in a full time capacity will have a minimum of one hour formal supervision/mentoring per fortnight or equivalent
- Allied health professionals who have practised for the equivalent of five years or more in a full time capacity will have a minimum of one hour supervision/mentoring per month or equivalent

The policy also recognises that many allied health professionals participate in more than the minimum requirements of professional support as outlined. NB: In some cases, greater amounts of professional support may be required to meet legislative, professional or individual requirements including:

- Learning and development programs such as the Allied Health Return to Practice (Re-entry) Program or the Rural and Remote Allied Health HP3 to HP4 Developmental Pathway Program
- Professional development pathways e.g. pharmacy professional development year, medical imaging professional development year, or provisional registration as a psychologist

**Links between Professional Support Activities and PAD**

Professional support activities are integrated into the Performance Appraisal and Development Plan. A Professional Support Plan is developed as a part of the Performance Appraisal and Development Plan (utilising part three of the Queensland Health template for PAD) which incorporates:

- Skills and knowledge required
- Goals
- Strategies for how to achieve goals
- Professional support and development options to be used

**Provision of Professional Support**

It is an expectation that allied health professionals with greater than two years’ experience will become professional support providers/supervisors as appropriate and as required. All supervisors will have access to support in their provision of supervision/mentoring and professional support activities. Profession specific managers will be consulted in determining the requirement for and appropriateness of staff to undertake professional support provision.

Legislative or other Authority

Related Policy or Documents

- Professional Support and Development Guidelines
- Allied Health Clinical Governance Framework
- HR-G Practice supervision in Allied Mental Health -
- Clinical Supervision Guidelines for Mental Health Services (Oct 2009)
- Queensland Plan for Mental Health 2007-2017
- HR- G 9 Performance Appraisal and Development - HR Policy G9
- Grievance Resolution HR Policy E-12
- Performance Improvement HR Policy - G11
- Ministerial Taskforce on Clinical Education and Training, 2007
- Allied Health Recruitment & Retention
- Forster Review
- Qld Health Clinical Governance Policy

Supporting Documents

## Review

This policy will be reviewed at least every two years.

Date of Last Review:

**Supersedes:** New policy

## Policy Custodian

Director, Allied Health Workforce Advice and Coordination Unit

## Responsible Executive Management Team member

## Approval and Implementation

## Approving Officer

## Approval Date

## Implementation Date

## Glossary of Terms used in this policy and supporting documents

| **Term** | **Definition** | **Source** |
| --- | --- | --- |
| **Professional Support** | A term that refers to activities that create an environment where personal and professional growth may occur | Steenbergen and Mackenzie, 2004:160. |
| **Professional Supervision** | A working alliance between two health professionals where the primary intention of the interaction is to enhance the knowledge, skills and attitudes of at least one of the health professionals. | Queensland Health, 2004 |
| **Peer Group Supervision** | A group that meets on a regular basis in order to review professional competence. | New Zealand Mentoring Centre, 2000 |
| **Profession Specific Manager** | This position promotes and leads their profession at a strategic level and most commonly refers to the Director of a profession in a facility or district. In situations where there is no Director, a profession-specific manager could be from an adjacent district/facility or division. | Queensland Health, 2010 |
| **Peer Review** | The presentation of a clinical scenario or case study to a group of peers where the ensuing discussion may validate current approaches to practice or provide ideas for alternate approaches. | Queensland Health, 2009 |
| **Mentoring** | A relationship which gives people the opportunity to share their professional skills and experiences, and to grow and develop in the process. Typically mentoring takes place between a more experienced and less experienced employee. | Office of the Director of Equal Employment Opportunity in Public Employment in Rural Connect: Mentor Handbook, 2001 |
| **Journal Club** | A group which reviews article/s relevant to allied health practice to ‘encourage reflection on clinical practice and an evidence-based approach to professional practice’. | Milinkovic et al 2008 |
| **In-Service** | A session where health professionals increase their professional knowledge and skills, and ensure they’re up-to-date with contemporary and evidenced based practices. | Queensland Health, 2009 |
| **Work Shadowing** | A method of professional support that involves engaging in a structured, goal directed learning placement in a work unit or area of practice in order to provide experience and contribute to the professional development of the participant. | Queensland Health, 2008 |

* Different professions/disciplines may use different definitions for the above terms as is appropriate for their workforce
